# Draft Genome Sequence of Toluene-Resistant *Staphylococcus epidermidis* SNUT

**DOI:** 10.1128/genomeA.00057-16

**Published:** 2016-03-03

**Authors:** Beomsoo Kim, Jingyu Kim, Hyun Park, Joonho Park

**Affiliations:** a Department of Fine Chemistry, Seoul National University of Science and Technology, Seoul, South Korea; b Korea Polar Research Institute, Incheon, South Korea

## Abstract

Here, we report draft sequence of the Gram-positive toluene-resistant bacterium *Staphylococcus epidermidis* SNUT. The draft genome sequence is 2,511,658 bases, with 2,346 protein-coding genes, 57 tRNA-coding genes, and 8 rRNA genes.

## GENOME ANNOUNCEMENT

It is known that organic solvents severely destroy membranes and inactivate enzymes of most bacteria (1), but some bacterial species are resistant to organic solvents. Mechanisms of solvent tolerance are well studied in Gram-negative bacteria but barely studied in Gram-positive bacteria. Most organic solvent-resistant bacteria have the structure of the Gram-negative bacterial cell wall, which has an additional outer membrane, allowing quick modifications and adaptations in lipopolysaccharides, efflux pumps, and/or fatty acid composition (2–4). However, mechanisms of organic solvent tolerance in Gram-positive bacteria have not been well studied (5).

The genus *Staphylococcus* is Gram positive, with a thick peptidoglycan layer in the cell wall, and it shows tolerance to organic solvents, such as toluene, benzene, and xylene. Still, the mechanisms of tolerance to organic solvents remain unclear (6, 7).

In our study, the toluene-resistant *Staphylococcus epidermidis* strain SNUT was isolated from a toluene-treated bioreactor. DNA extracted from a cell culture of *S. epidermidis* SNUT was subjected to whole-genome shotgun sequencing using the Nextera XT library preparation workflow (Illumina, San Diego, CA), and 2 × 300-nucleotide paired-end reads were generated on an Illumina MiSeq instrument. The reads were quality trimmed using the sliding window mode of the Trimmomatic program (8), with a cutoff value of 20. *De novo* genome assembly was performed using Celera Assembler 8.1. The draft genome sequence is 2,511,658 bases in length (444× coverage, 32% G+C content). Gene prediction and annotation were carried out using Glimmer3, the RAST annotation server, and the NCBI COG database. A total of 2,346 protein-coding genes, 57 tRNA-coding genes, and 8 rRNA genes were predicted in the draft genome. Approximately 82.9% of nucleotides were predicted to be protein-coding regions, and 1,854,897 bp (73.85%) of the protein-coding sequences were annotated with known proteins. A comparison with the genome sequences available with RAST showed that *S. epidermidis* ATCC 12228 (score, 537) and *S. epidermidis* RP62A (score, 525) were the closest neighbors of *S. epidermidis* SNUT. This result may provide further insights into the genetic determination of organic solvent resistance.

### Nucleotide sequence accession numbers.

This whole-genome shotgun project has been deposited in DDBJ/ENA/GenBank under the accession no. LQRB00000000. The version described in this paper is the first version, LQRB01000000.
